# Role of Reactive Oxygen Species in Cancer Progression: Molecular Mechanisms and Recent Advancements

**DOI:** 10.3390/biom9110735

**Published:** 2019-11-13

**Authors:** Vaishali Aggarwal, Hardeep Singh Tuli, Ayşegül Varol, Falak Thakral, Mukerrem Betul Yerer, Katrin Sak, Mehmet Varol, Aklank Jain, Md. Asaduzzaman Khan, Gautam Sethi

**Affiliations:** 1 Department of Histopathology, Post Graduate Institute of Medical Education and Research (PGIMER), Punjab, Chandigarh 160012, India; vaishali.pgi@gmail.com; 2 Department of Biotechnology, Maharishi Markandeshwar (Deemed to be University), Mullana, Ambala, Haryana 133207, India; thakralfalak@gmail.com; 3 Department of Pharmacology, Faculty of Pharmacy, Anadolu University, Eskişehir TR26470, Turkey; aysegulv@anadolu.edu.tr; 4 Department of Pharmacology, Faculty of Pharmacy, Erciyes University, Kayseri 38039, Turkey; eczbetul@yahoo.com; 5 NGO Praeventio, Tartu 50407, Estonia; katrin.sak.001@mail.ee; 6 Department of Molecular Biology and Genetics, Faculty of Science, Kotekli Campus, Mugla Sitki Kocman University, Mugla TR48000, Turkey; mehmetvarol@mu.edu.tr; 7 Department of Animal Sciences, Central University of Punjab, City Campus, Mansa Road, Bathinda 151001, India; aklankjain@gmail.com; 8 The Research Center for Preclinical Medicine, Southwest Medical University, Luzhou 646000, Sichuan, China; asadkhan@swmu.edu.cn; 9 Department of Pharmacology, Yong Loo Lin School of Medicine, National University of Singapore, Singapore 117600, Singapore

**Keywords:** reactive oxygen species (ROS), oxidative stress, inflammation, angiogenesis, metastasis, miRNA

## Abstract

Reactive oxygen species (ROS) play a pivotal role in biological processes and continuous ROS production in normal cells is controlled by the appropriate regulation between the silver lining of low and high ROS concentration mediated effects. Interestingly, ROS also dynamically influences the tumor microenvironment and is known to initiate cancer angiogenesis, metastasis, and survival at different concentrations. At moderate concentration, ROS activates the cancer cell survival signaling cascade involving mitogen-activated protein kinase/extracellular signal-regulated protein kinases 1/2 (MAPK/ERK1/2), p38, c-Jun N-terminal kinase (JNK), and phosphoinositide-3-kinase/ protein kinase B (PI3K/Akt), which in turn activate the nuclear factor kappa-light-chain-enhancer of activated B cells (NF-κB), matrix metalloproteinases (MMPs), and vascular endothelial growth factor (VEGF). At high concentrations, ROS can cause cancer cell apoptosis. Hence, it critically depends upon the ROS levels, to either augment tumorigenesis or lead to apoptosis. The major issue is targeting the dual actions of ROS effectively with respect to the concentration bias, which needs to be monitored carefully to impede tumor angiogenesis and metastasis for ROS to serve as potential therapeutic targets exogenously/endogenously. Overall, additional research is required to comprehend the potential of ROS as an effective anti-tumor modality and therapeutic target for treating malignancies.

## 1. Introduction

Despite continuous efforts in the development of novel treatment modalities, cancer still remains one of the most dreadful diseases in both developed and, developing countries; being an indomitable conundrum for scientists worldwide. Based on the recently published GLOBOCAN 2018 report, there was a prediction of 18.1 million new cancer cases and 9.6 million cases with cancer mortality in women and men. If the industrial development accompanied by environmental pollution and alterations in the human lifestyle, continue as fast as hitherto, predictions suggest by 2030, almost 13 million people will die from different cancers each year [1]. This would be a huge burden to the whole society and needs urgent intervention at different levels, including not only scientific contribution but also political statutes in implementation of diverse preventive measures.

The complexity of cancer is attributed to its multifaceted and multifactorial presentation. In the past few decades, the disruption of redox balance has been illustrated to be one of the most important reasons underlying cancer development, its progression, and metastasis in human cells [2]. This disproportionality in redox homeostasis is documented to be induced via increased free radicals, predominantly ROS. These highly active radicals originate from both intrinsic and extrinsic sources. Intrinsic sources of ROS mostly include mitochondria, inflammatory cells, and several enzymatic cellular complexes; extrinsic sources of ROS include pro-oxidant environmental toxins, radiation, [3] and diverse chemical compounds, including alcohol, tobacco smoking, and certain drugs [4]. Such free radicals can cause damage to various important biomolecules, including lipids, proteins, and nucleic acids, leading to oxidative stress and damaging different human tissues.

Elevated ROS levels, accompanied with down-regulation of cellular antioxidant enzyme systems, result in malignant transformation via different molecular targets, such as NF-κB and nuclear factor (erythroid-derived 2)-like-2 factor (Nrf2) [5,6]. Signaling cascades regulated by these key factors generate an inflammatory environment leading to the suppression of apoptotic cell death, tumor proliferation, angiogenesis, and metastasis; which cumulatively augment initiation, development, and progression of malignant neoplasms (Table 1). This review article summarizes the present up-to-date knowledge of the molecular mechanisms of ROS generation, its role in tumor growth and metastasis, along with highlighting the potential targets of the ROS cascade for potential therapeutic strategies.

## 2. Role of ROS in Cancer Progression

### 2.1. ROS-Mediated Induction of Oxidative Stress 

Oxygen is a multifaceted molecule, which is crucial for life sustainability but can be harmful when it is converted to ROS through oxidation-reduction reactions. The distinct aspect of ROS was first identified by Gerschmann in 1954 [20,21] and its free radical potential was described by Denham Harman in the free radical theory of aging in 1956 [21,22]. This initial work from the group; steadily triggered further investigations in the dimension of ROS to decipher their precise role in biological systems [21]. The discovery of the first described antioxidant enzyme, superoxide dismutase (SOD), by McCord [21,23] postulated the instrumental role of ROS through free radical generation. Following the initial studies on the substantial potential role of ROS, the focus shifted to delineate the beneficial and harmful effects of ROS in numerous pathological and physiological processes and their mechanism of action.

In a normal cell, ROS levels are balanced through numerous detoxification processes regulated through antioxidant enzymes. Hence, ROS homeostasis is well sustained, which contributes to the maintenance of redox balance in healthy cells. The complex I and III of the mitochondrial respiratory chain under high membrane potential, are contemplated to be the point of origin of ROS particularly, but numerous other resources may also play a pivotal role in elevated ROS generations, such as α-ketoglutarate dehydrogenase, monoamine oxidase, mitochondrial p66^Shc^, sirtuins, Nrf2, and forkhead box O3 (FOXO3) besides the underlying redox cycling reactions [24]. This elevated ROS production or defective defense mechanism may also lead to elevated oxidative stress levels leading to diverse pathological conditions. Examples of endogenous sources of ROS include mitochondrial oxidative phosphorylation, p450 metabolism, peroxisomes, and from the activation of inflammatory cells such as macrophages and neutrophils. It is thought that during the mitochondrial respiratory process, 1–2% of molecular oxygen is converted to ROS through one to three electron reductions and this leads to the formation of hydroxyl, hydrogen peroxide, superoxide, and peroxynitrite radicals [25].

**Figure 1:**
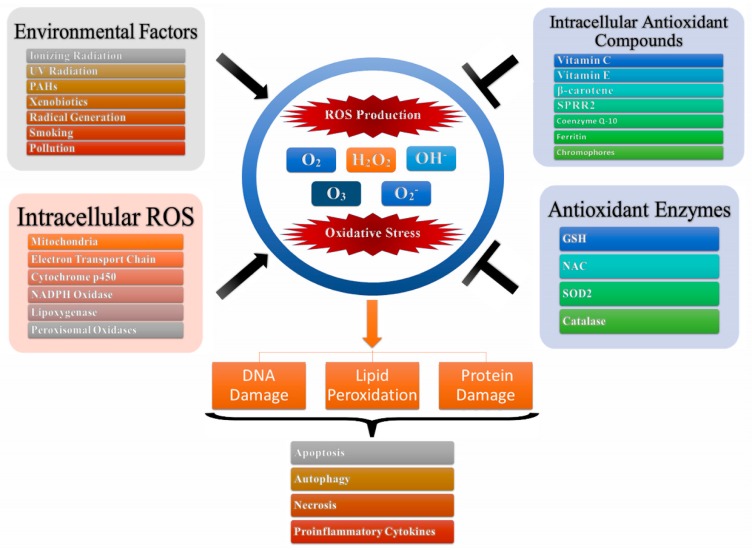
Oxidative stress and production of reactive oxygen species. Intracellular ROS and environmental factors (exogenous ROS) initiates ROS production leading to oxidative stress which in turn leads to DNA/lipid/protein degradation resulting in apoptosis, autophagy, necrosis and production of pro-inflammatory cytokines.

Oxidative stress is one of the main leading causes of toxicity which is attributed to the interactions of ROS as well as reactive nitrogen species (RNS) with cellular macromolecules such as DNA, lipid, and proteins, which interfere with the signal transduction pathways such as protein kinases, phosphatases, and transduction mechanisms (Figure 1). The pathological consequences of oxidative stress are characterized by impaired glucose tolerance due to mitochondrial oxidative stress. This is attributed to pro-oxidants changing their thiol/disulphide redox state which leads to diabetes mellitus and cancer or through the augmented action of either NAD(P)H oxidase leading to inflammatory oxidative conditions which is associated with chronic inflammation and atherosclerosis or through the action of xanthine oxidase-induced ROS formation which has been associated with reperfusion injury and ischemia [21]. Further, the ageing process can be attributed to the detrimental magnitude of free radicals leading to DNA damage, lipid peroxidation, and protein oxidation [21,22].

The connotation of oxidative and nitrosative stress with chronic and acute disease presentation is based on the biomarkers validated for oxidative stress. In an excellent review by Dalle-Donne and coworkers [21,26], the group summarized the biomarkers of oxidative stress and their correlation with human disease presentation. Natural antioxidants can protect the cell via scavenging the ROS. They may be separated into three classes—endogenous antioxidants (bilirubin, catalase (CAT), ferritin, SOD, glutathione, coenzyme Q, l-carnitine, alpha lipoic acid, glutathione peroxidase (GPx), melatonin, metallothionein, thioredoxins, peroxiredoxins, and uric acid), natural antioxidants (ascorbic acid, polyphenol metabolites, β-carotene, vitamin E, and vitamin A), and synthetic antioxidants (Nrf2, tiron, pyruvate, selenium, and *N*-acetyl cysteine (NAC)). Protecting the organism against harmful oxidants is a complex interaction between these antioxidants. High intracellular ROS concentration is important for damage, but another important fact is the equilibrium between ROS and antioxidant systems. The ROS production/antioxidant defense system balance is required for homeostasis [25]. Under normal conditions antioxidants outbalance oxidants but under oxidative conditions pro-oxidants prevail antioxidants [27].

### 2.2. Inflammatory Markers and ROS

**Figure 2:**
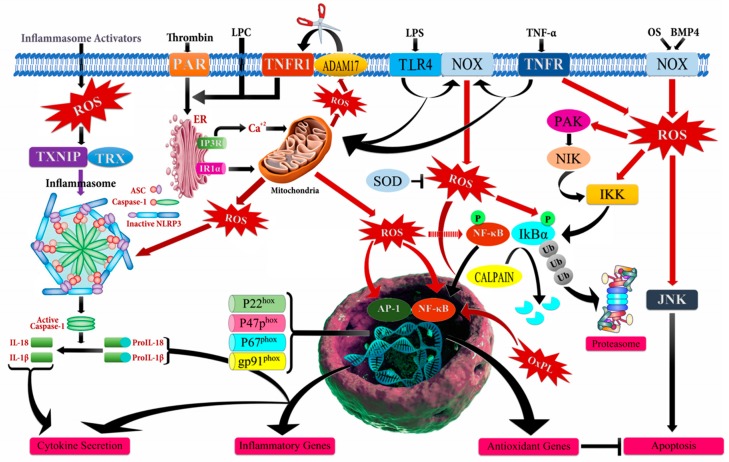
Schematic illustration of mechanism of action of reactive oxygen species (ROS) leading to inflammation. ADAM17 (ADAM metallopeptidase domain 17); ASC (Activating signal co-integrator 1); BMP4 (Bone morphogenetic protein 4); IKB-α (Inhibitor of nuclear factor kappa B kinase regulatory subunit alpha); IKK (Inhibitor of nuclear factor kappa-B kinase); IP3R (Inositol 1,4,5-trisphosphate receptor type 3); JNK (c-Jun N-terminal kinase); LPC (Lysophosphatidylcholine); LPS (Lipopolysaccharide); NF-кB (Nuclear factor kappa subunit B); NLRP3 (NLR family pyrin domain containing 3); NOX (NADPH oxidase); OxPL (Oxidized phospholipids); PAR (Par family cell polarity regulator); PAK (p21 (RAC1) activated kinase); SOD (Superoxide dismutase); TLR4 (Toll like receptor 4); TNF-α (Tumor necrosis factor alpha); TNFR (TNF receptor superfamily); TNFR1 (TNF receptor superfamily 1); TXNIP (Thioredoxin interacting protein); Ub (Ubiquitin).

The intricate relationship between cancer and, prolonged inflammation has been thoroughly investigated since Virchow’s hypothesis [28]. In 1863, Rudolf Virchow propounded that the “lymphoreticular infiltrate” reflected the origin of cancer at the locations of chronic inflammation [29]. The obtained epidemiological and experimental data from the numerous studies supported Virchow’s hypothesis and revealed that inflammatory processes regulate the course of cancer based on the level of inflammation-related factors, inflammatory cytokines, and chemokines, in the tumor microenvironment, either by producing an antitumor response or by inducing cell transformation and malignancy [30,31,32]. One of the major regulatory components in the relationship between cancer and chronic inflammation is ROS that has an ability to affect the type, presence, and levels of inflammation-modulating factors such as activator protein 1 (AP-1), β-catenin/Wnt (wingless related integration site), HIF-1α (hypoxia-inducible factor-1 alpha), NF-κB, PPAR-γ (peroxisome proliferator-activated receptor gamma), p53, inflammatory cytokines, chemokines, and growth factors [27,33,34,35]. However, it should be noted that there is a complex crosstalk between chronic inflammation, ROS accumulation, and cancer progression (Figure 2). The inflammatory cells in the tumor microenvironment lead to a massive production of ROS by activating the oxidant-generating enzymes such as inducible nitric oxide synthase (iNOS), myeloperoxidase (MPO), NADPH oxidase, and xanthine oxidase (XO), and by up-regulating cyclooxygenase 2 (COX2) and lipoxygenase (LOX) to remove and disrupt the biological, chemical, and physical factors. The abundant accumulation of ROS also produces oxidative damage to the DNA, RNA, proteins, lipids, and mitochondria.

This causes an increased mutation load, defects in signal-transduction, inactivation of apoptosis, and overpowered generation of additional ROS that activate the inflammation-modulating factors, inflammatory cytokines, and chemokines [34,35,36,37]. Apart from the induction of chronic inflammation by ROS-mediated NF-κB activation, the active NF-κB is considered to be a key component in the rise of therapy-resistant cancers toward fractional gamma-irradiation therapy and chemotherapeutic agents such as 5-fluorouracil, bortezomib, cisplatin, daunorubicin, doxorubicin, paclitaxel, vinblastine, vincristine, and tamoxifen, through the transcriptional up-regulation of Akt, Bcl-2 (B-cell lymphoma 2), Bcl-xL (B-cell lymphoma- extra-large), cyclin D1, COX-2, survivin, and XIAP (X-linked inhibitor of apoptosis) [38,39,40,41,42,43]. Targeting ROS consequently seems to be a very promising way to modulate cancer related chronic inflammation and the hallmarks for cancer development such as sustaining proliferative signaling, evading growth suppressors, activating invasion and metastasis, enabling replicative immortality, inducing angiogenesis, and resisting cell death [37,44].

### 2.3. Cancer Metastasis and ROS

**Figure 3:**
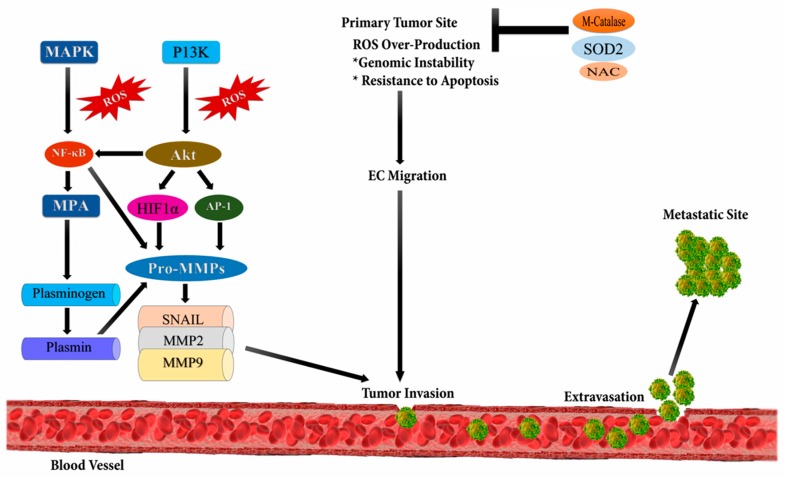
Reactive oxygen species and metastasis. High levels of reactive oxygen species leads to metastasis through the stimulation of phosphoinositide-3-kinase regulatory subunit/AKT serine/threonine kinases/mechanistic target of rapamycin kinase (PI3K/Akt/mTOR), and MAPK (Mitogen-activated protein kinases) signaling pathways which activates downstream SNAIL, MMP2 (metalloproteinase 2), and MMP9 (metalloproteinase 9) enzymes initiating epithelial-mesenchymal transition (EMT) leading to metastasis.

Metastasis involves the spread of cancer cells from the primary tumor to the surrounding tissues and to distant organs, and is the primary cause of morbidity and mortality [45]. Studies reveal that tumor metastasis is not an autonomous program but a complex and multifaceted event, occurring due to the intrinsic mutational burden of cancerous cells and bidirectional interaction between nonmalignant and malignant cells [46]. It occurs due to the up-regulation of several transcriptional factors such as NF-ĸB, ETS-1 (ETS proto-oncogene 1, transcription factor), Twist, Snail, AP-1, and Zeb (zinc finger E-box binding homeobox); metalloproteases viz. MMP-9 and, MMP-2; and chemokines or cytokines like transforming growth factor beta (TGF-β) (Figure 3) [47]. ROS plays an important role in the migration and invasion of cancerous cells. ROS are mainly produced as byproducts during mitochondrial electron transport in aerobic respiration and have numerous deleterious effects [48]. Epithelial to mesenchymal transition (EMT) is the major cause of tumor metastasis, where epithelial cells lose their polarity, cell-cell adhesion, and gain mobility.

Several studies have proved ROS to be a major cause of EMT. TGF-β1 regulates uPA (Urokinase type Plasminogen Activator) and MMP-9 to facilitate cell migration and invasion through ROS-dependent mechanisms [49]. Another study revealed that ROS increases tumor migration by inducing hypoxia mediated MMPs and, cathepsin expression [50,51]. According to a study reported by Zhang, NADPH oxidase 4 (NOX4)-dependent ROS production is necessary for TGF β1-induced EMT in MDAMB-231C and MCG-10A cell lines [52]. P53 also plays a major role in cell migration using cytokines TGF β1. Pelicano et al. suggested that mitochondrial dysfunction can lead to increased ROS production, which further up-regulates C-X-C motif chemokine 14 (CXCL14) expression through the AP-1 signaling pathway and, enhances cell mobility by elevating cytosolic Ca^2+^ levels [53]. ROS activate Nrf2 that stimulates Klf9 (Krupple like factor 9), thus activating ERK1/2; and results in an increased ROS production in cancer cells. Thus, a premalignant growth can be suppressed by using topical antioxidants that target Klf9 [54,55].

Mitochondrial Ca^2+^ also plays an important role in cancer metastasis. Jin and his colleagues observed that MCUR1 (Mitochondrial Calcium Uniporter Regulator 1) is up-regulated in hepatocellular carcinoma (HCC) which promotes EMT by activating ROS/Notch1/Nrf2 pathways. Thus, MCUR1 can be a potential target for the treatment of HCC [56]. Aydin et al. analyzed that NOX2 generates ROS, which influence metastasis by down-modulating the function of natural killer (NK) cells, and its inhibition can restore the IFNγ (interferon gamma)-dependent NK cell-mediated clearance of myeloma cells [57]. Vimentin protein also plays a major role in cancer initiation and progression such as EMT, and metastasis. Oxidative stress caused by HIF-1 regulates vimentin gene transcription, which helps in the formation of invadopodia during cancer cell invasion and migration [58]. Inhibition of vimentin expression by RNAi can reduce cell metastasis and hence decrease tumor volume [59]. ROS also induces epigenetic changes in the promoter region of E-cadherin and various other tumor suppressor genes, hence leading to tumor progression and metastasis. It causes hyper-methylation of the promoter gene by increasing Snail expression. Snail induces DNA methylation with the help of histone deacetylase 1 (HDAC1) and DNA methyltransferase 1 (DNMT1) [60].

### 2.4. Angiogenesis and ROS

**Figure 4:**
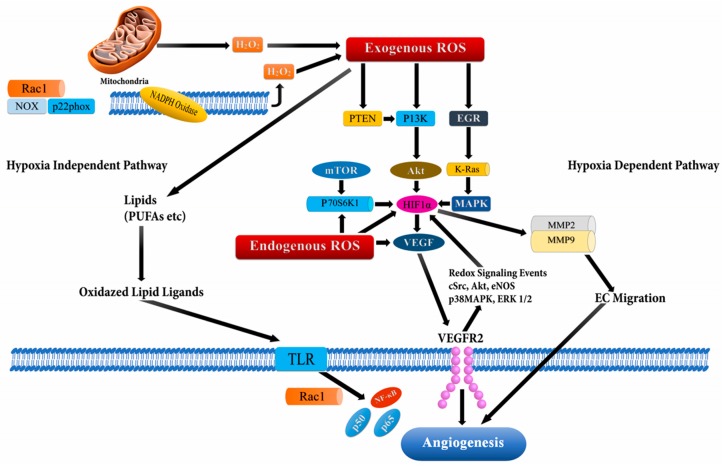
Angiogenesis activation through reactive oxygen species (ROS) via hypoxia dependent and hypoxia independent pathways. The hypoxia dependent pathway increases vascular endothelial growth factor (VEGF) expression via the phosphoinositide-3-kinase regulatory subunit/AKT serine/threonine kinases/mechanistic target of rapamycin kinase (PI3K/Akt/mTOR), PTEN (phosphatase and tensin homolog), and MAPK (Mitogen-activated protein kinases) signaling cascades via HIF-1α (Hypoxia-inducible factor1-alpha) and p70S6K1 (ribosomal protein S6 kinase B1), which release various cytokines, growth factors, and up-regulation of MMPs (matrix metalloproteinases) leading to angiogenesis. The hypoxia independent pathway leads to angiogenesis through oxidative lipid ligands which activates NF-кB (Nuclear factor kappa subunit B) via Toll-like receptors (TLRs).

During the initial stages of tumorigenesis, new blood vessels are formed from the pre-existing vasculatures, a process known as angiogenesis, which supports tumor proliferation and survival [61,62,63,64]. ROS-dependent angiogenesis is initiated through cancer proliferation, which in turn increases the metabolic rate leading to the generation of high ROS levels [63,64]. These elevated ROS levels lead to oxidative stress in the tumor microenvironment, which initiates secretion of angiogenic modulators [65]. Both endogenous and exogenous ROS spearheads stimulation of growth factors, cytokines, and transcription factors like VEGF and HiF-1α, which promote tumor migration and proliferation through ROS-dependent cellular signaling [62,65,66]. The signaling cascade through ROS mediation has been documented to perpetuate VEGF secretion and activate the PI3K/Akt/mammalian target of the rapamycin (mTOR) pathway through hypoxia independent or dependent mechanisms (through stabilization of HIF-1α which increases production of VEGF) (Figure 4), [67]. Additionally, the Ras signaling pathway has also been reported to up-regulate VEGF secretion [68]. Recently, mutant p53 was also recognized to modulate the angiogenic response in tumor proliferation through the ROS-mediated activation of VEGF-A and HiF-1 in HCT116 human colon carcinoma cells [69]. The mechanism of ROS-mediated angiogenesis has been extensively studied to understand the signaling cascade modulating cancer progression. In a study carried out using MDA-MB-231 breast cancer cells, deferoxamine (DFO) induced HIF-1α through ERK1/2 phosphorylation which promoted tumor migration and metastasis [70].

Han and colleagues demonstrated that the elevated levels of epidermal growth factor (EGF) triggered hydrogen peroxide production, which stimulated p70S6K1 via the PI3K/Akt signaling pathway, leading to the activation of downstream VEGF and HiF-1α [71]. On similar lines, Liu et al. reported that EGF lead to increased production of hydrogen peroxide in OVCAR-3 ovarian cancer cells, which activated the AKT/p70S6K1 pathway, thereby resulting in increased VEGF expression [72]. The group further documented that catalase overexpression and rapamycin inhibited angiogenesis. Hydrogen peroxide was also illustrated to inactivate phosphatase and tension homolog (PTEN) through the reversible oxidation of phosphatases in the cysteine thiol group and promote activation of the PI3K/Akt/mTORsignaling cascade and Ras [73].

In another study on ovarian cancer cells, Xia and colleagues documented that NOX4 knockdown lead to a reduction of VEGF and HIF-1α, which in turn regulated tumor angiogenesis [74,75]. A similar mechanism of action of ROS was illustrated through experiments in WM35 melanoma cells, where Akt induced the expression of NOX4 [75,76]. NADPH oxidase 2 (Nox2)-derived ROS was also reported to induce cancer progression and migration, modulated through the ERK/PI3K/AKT/Src (Proto-oncogene tyrosine -protein kinase)-dependent pathway leading to the activation of endothelial cells and induction of angiogenesis [77,78,79]. Nox1 (NADPH oxidase 1) was also reported to mediate the Ras-dependent up-regulation of VEGF expression and, angiogenesis via the Ras/ERK-dependent Sp1 phosphorylation and activation in CaCO-2 colon cancer cells [80,81]. In human umbilical vein endothelial cell (HUVECs), Angiopoietin -1 (Ang1) induced transient ROS through the activation of endothelial specific tyrosine kinase receptor, Tie-2, and p44/42, MAPK leading to vascular remodeling [82].

Moreover, copper was reported to increase the ROS-mediated expression of VEGF, HiF-1α, and G-protein estrogen receptor (GPER) in HepG2 hepatocellular carcinoma cells and SkBr3 breast cancer cells via activation of the EGFR/ERK/c-Fos pathway [83,84]. Similarly, the cadmium activated ERK/Akt pathway induced ROS expression and HiF-1; its downstream pro-angiogenic molecule in BEAS-2B bronchial epithelial cells [85]. In addition, several other extracellular remodeling proteins and transcription factors (p53, HiF-1α, VEGF, and MMPs) have been documented to be regulated by ROS [86,87,88,89,90,91]. Also, numerous studies in cancer cell lines (MCF-7, HepG2, H-1299, PC-3) showed that ROS acted through the PI3K/Akt signaling cascade, thereby enhancing HiF-1α expression and angiogenesis [92,93,94]. In view of literature supporting the angiogenic potential of ROS, therapeutic targets with antioxidant potential and targeting subsequent signaling cascades will be of clinical significance in the management and treatment of patients through down-regulation of neovascularization. Different strategies to restore redox imbalance is another avenue to improve outcome of diseases. As the interventional trials with small anti-oxidants have not been effective, further research to explore disease-specific ROS may be of clinical relevance for futuristic drug development.

## 3. Role of ROS in Cancer Cell Killing

### 3.1. Cellular Apoptosis and ROS

**Figure 5:**
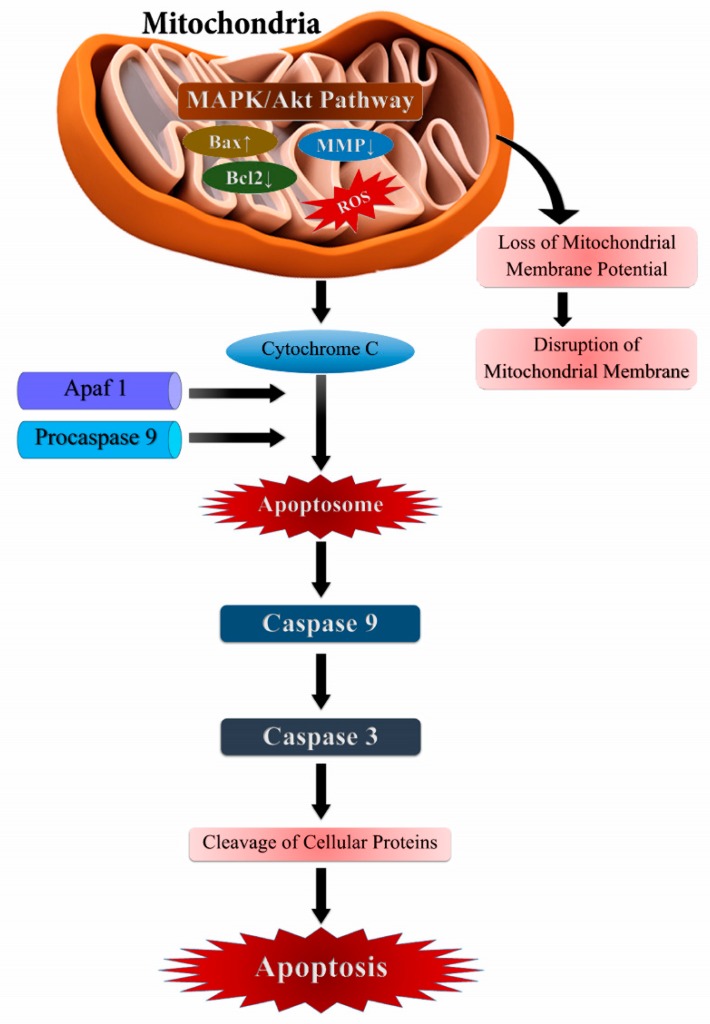
Exogenously or endogenously produced reactive oxygen species (ROS) activates extrinsic and intrinsic apoptosis pathways. ROS modulated cell-signaling activation of MAPK (Mitogen-activated protein kinases), Bcl_-_2 (BCL2 Apoptosis Regulator), and Bax (BCL2 Associated X, Apoptosis Regulator) which activates the downstream caspase cascade, leading to apoptotic cell death.

Almost all forms of DNA damage, like base modifications, strand breakage, DNA cross-linking, and proteins are induced by ROS, which are associated with cancer initiation and development [95]. However, a paradox in biological systems is that ROS can induce apoptotic cell death, which is an important approach in cancer therapeutics [96,97,98]. ROS disrupts the mitochondrial membrane and opens the mitochondrial permeability transition pore (PTP), and thus interferes with mitochondrial electron transfer chain and induces the release of Cytochrome-c. In the cytosol, together with Apaf-1 (apoptotic peptidase activating factor 1) and procaspase-9, Cytochrome-c forms ‘apoptosomes’ leading to the activation of caspase-9, which then activates effector caspases, e.g. caspase-3, that results in the cleavage of cellular proteins and apoptotic cell death (Figure 5) [99,100,101,102]. Indeed, hydrogen peroxide (H_2_O_2_) is one of the most important among the ROS group which is a direct and potent inducer of apoptosis [103].

Numerous studies [104,105,106,107,108] have illustrated that anticancer agents induce cancer cell apoptosis and autophagy via ROS generation as detailed in Table 2. For example, natural polyphenol resveratrol was found to induce mitochondrial accumulation of H_2_O_2_ by regulating antioxidant enzymes, which in turn, induced apoptosis in PC-3 (prostate cancer), HepG2 (hepatic cancer), and MCF-7 (breast cancer) cells [109]. Some evidence have also shown that the black cumin component, thymoquinone, also act as a pro-oxidant and induces apoptosis by generating ROS through different molecular pathways, like activating Akt and causing conformational changes in BCL-2 associated X, apoptosis regulator (Bax) protein, which leads to the loss of mitochondrial membrane potential and release of cytochrome-c and subsequent activation of the caspase-dependent apoptotic pathway [110,111,112,113,114]. Several studies have also discovered that ginsenosides exert their anticancer activities by generating ROS via a signaling cascade, where ROS was found to be responsible for inducing apoptosis [115].

### 3.2. Autophagy and ROS

The process of autophagy, involving degradation of organelles and proteins, plays a crucial role in cellular processes which is associated with elevated ROS levels. Numerous studies have documented the role of ROS regulation in the mediation of autophagy [127,128,129]. The diverse effects of autophagy range from prevention of infection to pathogen elimination to cell death of dysfunctional cellular organelles. These consequences indicate the potential of ROS to act as a signaling target in survival related to autophagy [130,131,132]. Recently, research has transitioned into looking for the potential applicability of ROS-derived autophagy in the treatment of malignancies [133,134,135]. The intracellular ROS levels have also been reported to directly corroborate with the regulation of autophagy induction in malignant tumors [136,137]. Autophagy related 4A cysteine peptidase (ATG4) enzyme oxidation, a prerequisite for de-lipidation of the ATG8 protein, induced by H2O2 leads to the induction of autophagy. This H2O2-induced oxidation in turn inactivates ATG4 which subsequently results in an elevated LC3-associated autophagosomes production [136]. On the other hand, the AMP-activated protein kinase (AMPK) pathway also plays a major role in the ROS related autophagy regulation. This activation of AMPK inhibits mTORC1 resulting in autophagy induction. Additionally, oxidative stress also regulates AMPK pathway activation via phosphorylation of AMPKK (AMPK kinase) which increases H2O2 production, thereby resulting in apoptosis induction [136]. In addition, various transcription factors e.g., NF-κB can also modulate the expression of autophagy-associated genes (ATG6/BECLIN1 or p62/SQSTM1) which directs ROS induced autophagy in cancer [138,139]. In an initial study by Kim and colleagues, SOD overproduction was documented to inhibit selenite induced cytotoxicity (autophagy) in human glioma cells [140]. Additionally, small miRNA was reported to knockout ATG6/7 (Autophagy related gene 6 and 7), which in turn reduced selenite induced cytotoxicity [132,141,142]. Based on these findings, it has been postulated that elevated ROS levels and its modulation induces autophagy in malignant cells.

### 3.3. Anticancer Therapy and ROS 

ROS concentration in tumor cells is very critical in anticancer therapy. High ROS levels are documented to induce cytotoxicity along with reversal of chemotherapeutic resistance in tumorigenic cells. Several investigations are suggestive of the fact that elevated ROS levels is the underlying core mechanism of action and efficacy of conventional cancer therapies which is directly associated with cancer cell death [143,144,145,146]. The mechanism of elevated ROS levels to target malignant cells is either via augmenting ROS generation through exogenous agents or inhibitors of the antioxidant system.

### 3.4. Inhibition of Antioxidant System in Cancer Cells 

The disruption or inhibition of cellular antioxidant enzymes augment ROS production which trigger apoptosis in malignant cells. SOD, Glutathione (GSH) system, and Thioredoxin 1 (Trx) are the major targets of ROS enhancing antitumor agents [142,147,148]. In a normal healthy cell, ROS levels are low which signify their lower dependence on antioxidant enzymes. Numerous studies have reported the findings that inhibition of cellular antioxidant system lead to the induction of ROS-mediated cytotoxicity (autophagy) in different types of cancers [147,149,150,151,152]. Further, elevated cellular antioxidant levels are also reported to be directly involved in developing chemoresistance in malignant cells. To overcome chemotherapy resistance, many chemotherapeutic compounds and antitumor agents have been developed which specifically target intracellular GSH levels. For example, administration of PEITC (phenethyl isothiocyanate) in combination with GSH alleviates the GSH pool, subsequently stemming oxidative stress leading to cytotoxicity in malignant cells. This was further reported to block GPX in HRAS-transformed ovarian carcinoma cells, thereby inducing cell death [149,153,154]. In another study on similar lines, β-PIETC was documented to block GPX thereby decreasing GSH levels which induced high ROS expression in tumor cells [155,156,157]. Hence, targeting and reducing GST and GSH levels is illustrated to restore the cytotoxic ability of various arsenic derivatives, alkylating agents, and platinum derived agents. Buthioninesulfoximine (BSO) has been identified to halt GSH production and is being increasingly studied owing to its high sensitivity [158,159,160]. ATO (Arsenic trioxide) and BSO administration in acute promyelocytic leukemia (APL) was reported to synergistically reduce GSH levels thereby inducing apoptosis [161,162,163,164]. Similarly, copper *N*-glycinate (CuNG), a copper derivative, has been illustrated to target GSH in Ehrlich ascites carcinoma and stimulate ROS production [164,165,166].

In addition to GSH, SOD1 can also be targeted specifically to induce apoptosis in different types of cancers. This SOD inhibition has presented as a promising approach for targeting tumor cells. In an in vitro study carried out on lung adenocarcinoma, inhibition of SOD1 alleviated the growth of KRAS-mutant tumor cells [147,167]. Similarly, in another study, methoxyestradiol (2-ME), was illustrated to block SOD, which lead to the induction of cell death in leukemia cells. NRF2, is also a key regulator of cellular antioxidants and KRAS and MYC oncogenes have been reported to mediate NRF2 transcription. Hence, NRF2 can be therapeutically targeted to stimulate ROS-induced apoptosis in tumor cells [168,169].

Additionally, Trx and TrxR is also an appropriate target for the development of novel antitumor treatments. This is attributed to augmented Trx and TrxR levels reported to positively correlate with tumor progression, chemo resistance, and poor survival [170,171,172] Presently, several compounds specially targeting Trx are being explored on the scientific front in pre-clinical and clinical models. For instance, a Trx-blocker, motexafin gadolinium, in phase III clinical trials has shown to specifically target malignant cells [173,174].

### 3.5. Production of ROS Directly in Cancer Cells 

Inhibition of antioxidant enzymes is another therapeutic approach in the induction of ROS production in tumor cells [143,175]. Numerous approaches have been developed to administer ROS and ROS producing agents in vitro in cell cultures, which would enhance ROS levels and ultimately cause cellular damage [176,177]. Some of these compounds have been approved as anticancer drugs and many are still being developed. These drugs/agents are either used alone or in combination with chemotherapy and/or radiotherapy. Of these, some chemotherapeutic agents like antifolates, alkaloids, and taxanes, disrupt the mitochondrial electron transport chain (ETC), thereby resulting in high O2− levels and the induction of mitochondria-mediated cell cytotoxicity [178,179,180]. Other chemotherapeutic agents like doxorubicin and cisplatin also lead to increased ROS production, levels of which are cytotoxic to tumor cells.

Procarbazine was the first ROS inducing drug used in anticancer therapy [181]. Procarbazines lead to the production of azo-derivatives, which in turn lead to ROS generation and oxidative DNA damage. In 1963, the first clinical trial of Procarbazine was conducted following which it was approved as a cytotoxic agent for treatment of brain tumors and Hodgkin’s lymphoma [182,183,184]. In last span of 15 years, numerous conventional antitumor agents inducing ROS as a cancer therapeutic modality has been extensively investigated for their safety and efficacy. For instance, doxorubicin and anthracyclines induce ROS production and are widely used in the treatment of acute lymphocytic leukemia (ALL), bladder cancer, lymphoma, Kaposi’s sarcoma (KS), breast cancer, and other malignancies [185,186,187,188]. In addition, biological molecules can also stimulate ROS generation leading to apoptotic cell death. ROS-dependent arsenic drugs are reported to be used in APL treatment [178,189]. Also, Imexon has shown to elevate oxidative stress and stimulate apoptosis in cancer cells. In preclinical investigations and phase I/II clinical trials, the safety and anticancer effect of Imexon has been very well studied in leukemia [190].

Several anticancer drugs have also been reported to disrupt the ETC, resulting in elevated ROS production [179]. Further, these ROS-generating agents directly target the complex I/II of ETC [175,180]. For example, ATO has been successfully used for the treatment of APL patients, wherein ATO leads to the generation of ROS via NADPH oxidase, which in turn induces apoptotic cell death [178,189].

Some studies have also indicated that administration of therapeutic agents increasing ROS production along with conventional chemotherapies, have shown increased efficacy in targeting tumor cells [175,191,192]. The synergizing effect of emodin and ROS-inducing therapeutic agents have been documented to induce cell apoptosis in different types of cancers [192,193,194]. Further, the safety was also monitored and was shown to cause negligible damage to healthy cells.

### 3.6. miRNAs and ROS

**Figure 6:**
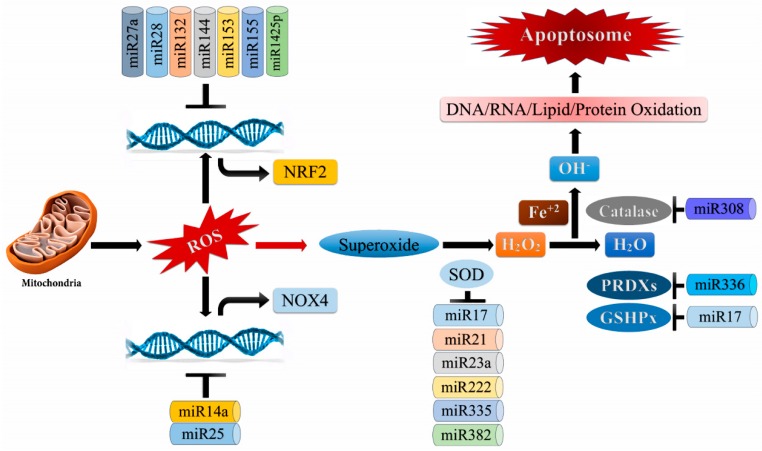
Regulation of microRNA biogenesis through reactive oxygen species (ROS): Complex I/III in mitochondria leads to elevated superoxide anion (O_2_^-^) production. Superoxide dismutase (SOD) converts O_2_^-^ to H_2_O_2_ (hydrogen peroxide), which is acted upon by glutathione peroxidase (GSHPx)/ catalase (CATs) and converted to H_2_O (water). H_2_O_2_ leads to Hydroxyl radicals (OH) production leading to DNA/RNA/lipid/protein degradation.

As both reactive oxygen species and microRNAs (miRNAs) are dysregulated in cancers, it is imperative to understand their association, and how they maintain homeostasis and avert tumorigenic conversion from a healthy cell. In view of reported scientific literature, accumulative evidence is suggestive of the crosstalk between ROS signaling and miRNAs expression. miRNAs are a class of small endogenous non-protein coding RNAs that have the size of approximately ~22 nts; they are capable of altering the expression of target genes at the post-transcriptional level, mainly by binding at the 3′UTR region of their mRNA [195]. In this section, we are going to discuss the latest findings on ROS-mediated regulation of miRNAs, ROS pathways regulated by miRNAs in cancer, and the ROS-miRNAs axis as a potential target for cancer as illustrated in Figure 6.

In an interesting study by Yang et al., 2019, [196], the group reported that the deprivation of glutamine in pancreatic ductal adenocarcinoma (PDAC) cells can lead to significant up-regulation (~3-fold) of miR-135a and miR-135b expression as compared to PDAC cells having glutamine [196]. Further, it was shown that when PDAC were treated with an antioxidant *N*-acetyl-L-Cysteine glutamine molecule, the levels of miR-135a and miR-135b decreased, which suggested that the induction of the miR-135 family is ROS-dependent. Glutamine is known to regulate the ROS level in the cells [197]. Mechanistically, it was demonstrated that low levels of glutamine in cells induced ROS species activation, which in turn activates mutant p53 and increases the expression of miR-135 by binding to its promoter [196].

Another group have shown that when hepatocellular carcinoma cells (HepG2) were exposed to H_2_O_2_ there was a significant decrease (~1.5 fold) in the expression of miR-145 and miR-128 levels in the cells [198]. Further, the authors have demonstrated that the insulin-induced ROS is responsible for the decrease in miR-145 and miR-128 expression in cells, and the overexpression of either miR-145 or miR-128 eliminates insulin-induced ROS and pyruvate kinase M2 expression. It was also shown that when vascular cells were exposed to a high concentration of H_2_O_2_, the expression of miR-200c up-regulated, which further leads to cellular apoptosis and senescence through binding of the ZEB transcription factor 1 (ZEB1) [199]. Normally miR-200c acts as a tumor suppressor in the bladder, gastric, and ovarian cancers. Similarly, another group reported a group of four miRNA signatures namely, miRNAs let-7s, miR-34s, miR-200s, and miR-182, which were significantly increased in high-grade serous ovarian carcinoma cells post-H_2_O_2_ exposure [200]. They further demonstrated that ROS up-regulates β-catenin, which in turn regulate the expression of miR-182 in cells. He et al., 2012 have demonstrated that via epigenetic mechanisms, ROS decreases miR-199a and miR-125b expression in ovarian cancer cells, through the promoter methylation of miR-199a and miR-125b [201]. It was also documented that Chidamide, a HDAC inhibitor (HDACi), increased ROS production in cells and up-regulated miR-129-3p expression in H1355 and A549 lung adenocarcinoma cells [202]. In cells, Chidamide suppressed telomerase activity through ROS accumulation and cell cycle arrest.

In addition to acting as an essential target of ROS-mediated stress molecules, miRNAs are also found to control genes that are either ROS activators or scavengers. For example, miR-9 suppressed the glutamic-oxaloacetic transaminase (*GOT1*) gene by directly binding to its 3’-UTR in melanoma cells, which subsequently abridged erastin- and RSL3-induced ferroptosis. Ferroptosis is a form of cell death process driven by lipid-based ROS accumulation [203]. Another study showed that miR-34a directly targets the *NOX2* gene and induces apoptosis in glioma cells through NOX2-derived ROS generation [204]. The NOX2 subunit is the catalytic core of the NADPH oxidase complex, which is considered as the major source of ROS production in epithelial cells and increases cancer risk [205,206]. Similarly, miR-23b was reported to down-regulate proline oxidase expression by directly targeting its 3′UTR and thereby promoting renal cancer [207]. The proline oxidase gene is a mitochondrial tumor suppressor gene that is known to induce apoptosis through ROS production and also reduces HIF [208]. He et al., 2018 have demonstrated that the miR-422 a-pyruvate dehydrogenase kinase 2 axis influence de novo lipogenesis in gastric cancer cells, that subsequently increases ROS production and rapid hypo-phosphorylation of retinoblastoma protein and finally cell cycle arrest at the G1 phase of the cell cycle [209]. Furthermore, another group have shown that miR-148b suppressed cell proliferation and regulated the oxidative stress response in human endometrial cancer RL95-2 cells by decreasing the expression of HIF-1α and nuclear factor erythroid 2-related factor by down regulating the endoplasmic reticulum MMP1 gene [210].

From the above-mentioned studies, it is very clear that both ROS and miRNAs are interlinked to each other and play an important role in the pathogenesis of cancer. Hence, the future targeting of ROS with miRNAs inhibitors may represent a novel therapeutic approach for the treatment of cancer.

## 4. ROS: A Double-Edged Sword

In a normal cell, redox homeostasis is sustained amidst ROS production and exclusion due to the conserved antioxidant mechanism via enzymes (glutathione peroxidase, superoxide dismutase, and catalase) and transcription factor Nrf2 [211]. Excessive ROS generation leads to a defective antioxidant defense mechanism, incompetent to scavenge excess, thereby leading to impaired balance between antioxidants and pro-oxidants. Recent literature has emphasized the dichotomous nature of ROS in malignant cells, depending on the stage of cancer progression i.e., early stage/late stage, on the basis of which differential effects of ROS are reported in tumor cells. In a tumorous cell, elevated ROS production initiates an adaptation reaction, which subsequently maintains the redox balance. At the precancerous/early stage of tumor progression, moderate ROS levels induce tumorigenesis, tumor promulgation, metastasis, and survival [211]. With tumor progression, elevated ROS levels beyond the toxic threshold lead to cell death, apoptosis [212,213], and senescence [212]. Through the inclusion of dietary antioxidants i.e., phytochemicals, the level of cellular antioxidants can be regulated, which can in turn regulate the growth inhibition and cell death in malignant cells. In MCF-7 breast cancer cells, tamoxifen-induced cytotoxicity was reported to be regulated via the intracellular concentration of vitamin C, which inhibited lipid peroxidation leading to decreased ROS levels [214]. In addition, in MDA-MB-435, SKBR-3, and MDA-MB-231 cells, resveratrol reduced ROS accumulation which was shown to decrease paclitaxel-induced cell death [215,216]. Vitamin E was also reported to decrease ROS production in a dose-dependent manner in a MCF-7 orthotropic breast tumor model. The results from the study showed decreased ROS levels post-12 days treatment followed by tumor growth in breast cancer cells and p53 expression [217]. On the other hand, phytochemicals like vitamin C, resveratrol, apigenin, luteolin, and epigallocatechin-3-gallate etc. have been reported to have pro-oxidant effects leading to elevated ROS levels and cell death. In an in vitro study, elevated vitamin C doses have been reported to induce pro-oxidant activity via high H_2_O_2_ generation [218]. In MCF-7 breast cancer cells and HT29 colon cancer cells, vitamin C-induced oxidative stress can lead to NAD depletion and inhibition of energy metabolism causing cellular apoptosis [219]. Resveratrol was also seen to have pro-oxidant activity resulting in elevated formation of hydroxyl radicals in the presence of copper ions [220,221]. These phytochemicals are being increasingly explored for the development of ROS-targeted killing of tumor cells as anticancer therapeutic agents.

## 5. Conclusions and Future Perspectives

For the healthy functioning of normal cells, a delicate balance in ROS redox processes, which maintains physiological homeostasis, is critical. This is elicited in numerous in vitro and in vivo studies emphasizing the significant role of ROS homeostasis in cellular signaling. ROS also plays a pivotal role in cellular processes, with low levels playing a substantial role in the regulation of signaling cascades while higher concentrations of ROS initiates apoptosis/cell damage. The decrease in effective neutralization of excessive ROS can result in different serious diseases, such as cancer. The aberrant production of ROS leads to cancer growth and progression via different signaling pathways (PI3/Akt/mTOR, PTEN, MAPK, VEGF/VEGFR, and MMPs). However, the substantial increase in ROS levels has been shown to intervene in processes important for cancer progression, initiate apoptosis, and contribute to cell death induced by traditional therapeutic modalities, including chemotherapy. A number of chemotherapeutic drugs have been reported to induce apoptosis through ROS. As ROS have diverse molecular targets in cells, their activities are also regulated by a wide range of signaling pathways. ROS is also known to cause drug resistance to chemotherapeutic treatments. It acts through the activation of NF-κB, which triggers the secretion of pro-inflammatory cytokines. Hence, the dual role of ROS should be exploited as a therapeutic target to inhibit tumor growth via impeding inflammation, angiogenesis, and metastasis. To develop an effective anti-tumor treatment modality, the scientific groups need to completely comprehend ROS and its redox state in malignancies, which is still enigmatic despite extensive research.

## Figures and Tables

**Table 1:** Role of ROS in cancer progression.

Effect	Mechanism	Cell Line	References
Oxidative Stress	Aquaporin AQP5-mediated H_2_O_2_ influx rate indicates the presence of a highly efficient peroxiporin activity and consequently activates signaling networks related to cell survival and cancer progression	Pancreatic Carcinoma line-3 (BxPC3)	[7]
	PCB118 promotes hepatocellular carcinoma cell (HCC) proliferation via Pyruvate kinase M2 (PKM2)-dependent up-regulation of glycolysis, which is mediated by Aryl hydrocarbon receptor/Nicotinamide adenine dinucleotide phosphate oxidase (AhR/NADPH oxidase)-induced ROS prouction	SMMC-7721	[8]
	Enhanced ROS of exposed cells alters the mitochondrial metabolic activities in terms of increased mitochondrial mass and DNA content and initiates cancer progression through modifying cellar biomarkers	MOE1A	[9]
Inflammatory markers	Serum ROS and damaged mtDNA may be markers of mitochondrial metabolism through oxygenation of the primary tumor and results in systemic inflammation and adverse outcomes of locally advanced rectal cancer (LARC)	HCT-116, HT-29, and LoVo	[10]
	Inflammation in the stroma induces TNF-α signaling and the NOX1/ROS signaling pathway is activated downstream with expression of TLR2 which is an important tumor-promoting mechanism stimulated by inflammation	Mouse Model	[11]
	Alkylating agents may evoke inflammatory responses that seem to contribute to malignant progression in specific breast cancer cells	MDA-MB231, Hs578T, SKBR3 and MCF7	[12]
Metastasis	ROS induce epithelial-mesenchymal transition (EMT), the glycolytic switch, and mitochondrial repression by activating the Distal-less homeobox-2 (Dlx-2)/Snail axis, thereby playing crucial roles in metastasis	MCF-7	[13]
	Elevated mitochondrial ROS via fatty acid β-oxidation, activates the MAPK cascades, results in EMT process of ROS high tumor spheres (RH-TS) cells, and enhances metastasis	4 T1, SW480, HCT116 and HT29	[14]
	Loss of TMEM126A induces ROS production with mitochondrial dysfunction and subsequently metastasis by activating extracellular matrix (ECM) remodeling and EMT	MDA-MB-231HM	[15]
	PM2.5 exposure induces ROS, which activates loc146880 expression and promotes the malignant behavior	A549	[16]
Angiogenesis	ROS-ERK1/2-HIF-1α-VEGF-induces angiogenesis by increased level of RRM2	C33A and MCF-7	[17]
	High glucose increases angiogenesis and decreases apoptosis due to activation of the NF-κB pathway by increasing ROS	MCF-7	[18]
	27-Hydroxycholesterol (27HC) enhanced the generation of ROS and activates the STAT-3/VEGF signaling in an ER independent manner which results in induced angiogenesis	Breast Cancer Cells	[19]

**Table 2:** Role of ROS in cancer cell death.

Effect	Mechanism	Cell Line	References
Apoptosis	Increase in cell oxidation by c-Met-Nrf2-HO-1 pathway and promotes apoptotic cell death	786-O and ACHN	[116]
	Apoptosis enhanced by ROS by affecting MAPK & AKT signaling and DNA damage mediated p53 phosphorylation	HePG-2 Cells	[117]
	↓ ROS by expression of GPx3 and leads to G2/M arrest	H157, H460, A549, H1299, H1650, and H1975 lung cancer cells	[118]
	↑ ROS by knockdown of nicotinamide nucleotide transhydrogenase and significant cell apoptosis under oxidative Stress	GES-1, SGC7901, SNU216, MKN45, MKN74, BGC823, HGC27 and MGC803	[119]
Short mRNA	Salviamiltiorrhiza treatment induces apoptosis through regulation of miR-216b and ROS/ER stress pathways	U266 and U937 Cells	[120]
	miR-21 silencing effect the ROS-induced activation, invasion, migration, and glycolysis of Pancreatic stellate cells (PSCs)	Human PSCs, Panc-1	[121]
	Down-regulation of NOX2 using siRNA technology in decreased cell viability and ROS content	SNU719 cells	[122]
	Melanoma differentiation-associated gene-7/interleukin-24 (*mda-7/IL-24*) regulates miRNA biogenesis through alteration of ROS-dependent MITF-DICER pathway	Animal cancer model	[123]
Autophagy	Silencing of YAP enhanced autophagic flux by increasing RAC1-driven ROS, through inactivation of mTOR	BEL/FU, BEL-7402	[124]
	Zinc Oxide Nanoparticle (ZON) evoked autophagy by accelerating the intracellular dissolution of ZONs and ROS generation.	MCF-7/ADR	[125]
	Cell killing was due to the summative effect of caspase-dependent intrinsic apoptosis and caspase-independent autophagy by activation of MAPK family members (ERK1/2 and JNK) with generation of ROS	SNU-719	[126]
